# Molecular Epidemiology and Disease Severity of Human Respiratory Syncytial Virus in Vietnam

**DOI:** 10.1371/journal.pone.0045436

**Published:** 2013-01-22

**Authors:** Dinh Nguyen Tran, Thi Minh Hong Pham, Manh Tuan Ha, Thi Thu Loan Tran, Thi Kim Huyen Dang, Lay-Myint Yoshida, Shoko Okitsu, Satoshi Hayakawa, Masashi Mizuguchi, Hiroshi Ushijima

**Affiliations:** 1 Department of Developmental Medical Sciences, School of International Health, Graduate School of Medicine, The University of Tokyo, Tokyo, Japan; 2 Department of Pediatrics, University of Medicine and Pharmacy at Ho Chi Minh City, Ho Chi Minh City, Vietnam; 3 Children's Hospital 2, Ho Chi Minh City, Vietnam; 4 Institute of Tropical Medicine, Nagasaki University, Nagasaki, Japan; 5 Division of Microbiology, Department of Pathology and Microbiology, Nihon University, School of Medicine, Tokyo, Japan; University of Iowa, United States of America

## Abstract

Respiratory syncytial virus (RSV) is a major cause of acute respiratory infections (ARIs) in children worldwide and can cause high mortality, especially in developing countries. However, information on the clinical and molecular characteristics of RSV infection in developing countries is limited. From April 2010 to May 2011, 1,082 nasopharyngeal swabs were collected from children with ARI admitted to the Children's Hospital 2, Ho Chi Minh City, Vietnam. Samples were screened for RSV and genotyped by reverse transcription-PCR and sequencing. Demographic and clinical data was also recorded. RSV was found in 23.8% (257/1,082) of samples. RSV A was the dominant subgroup, accounting for 91.4% (235/257), followed by RSV B, 5.1% (13/257), and 9 cases (3.5%) were mixed infection of these subgroups. The phylogenetic analysis revealed that all group A strains belonged to the GA2 genotype. All group B strains belonged to the recently identified BA genotype, and further clustered into 2 recently described subgenotypes BA9 and BA10. One GA2 genotype strain had a premature stop codon which shortened the G protein length. RSV infection was significantly associated with younger age and higher severity score than those without. Co-infection with other viruses did not affect disease severity. RSV A caused more severe disease than RSV B. The results from this study will not only contribute to the growing database on the molecular diversity of RSV circulating worldwide but may be also useful in clinical management and vaccine development.

## Introduction

Respiratory syncytial virus (RSV) is the major cause of acute respiratory infections (ARIs) among infants and young children worldwide [1]. The clinical presentations can vary from mild upper respiratory tract infections (URTIs) to life threatening bronchiolitis and pneumonia which result in significant pediatric hospitalization and economic burden [2]. Primary RSV infections occur during the first year of life in more than 50% of infants, and by 2 years of age, almost all children have been infected at least once [3]. RSV can cause re-infections throughout life with milder disease, indicating that either RSV infection induces an inadequate immune response or genetic variability of RSV is extensive [4]. RSV is a negative-sense single-stranded RNA virus that belongs to the *Paramyxoviridae* family. RSV is divided into two major groups, A and B, initially based on the reaction of the virus with monoclonal antibodies against the major structural glycoproteins G and F [5] and later by genetic analysis [6]. Each group can be further subdivided into genotypes by nucleotide sequence variability. The attachment glycoprotein G is the most divergent viral protein, both between and within the two groups, and a major target for neutralizing and protective antibody responses [7].

Along with the F protein, the second variable region at the C-terminal of the G protein that contains much of the G gene variability is commonly used in molecular epidemiological studies [8]. So far, RSV group A is divided into 8 genotypes (GA1 to GA7, and SAA1), and so is RSV group B (GB1 to GB4, SAB1 to SAB3, and BA) [9]. BA, which was first isolated in Buenos Aires in 1999, is a new genotype of group B with a 60-nucleotide duplication in the second variable region of the G protein gene [10]. The two groups circulate independently, but often at the same time, although group A viruses tend to predominate [3], [11]. The presence of two groups has led to the speculation that there might be a relationship between the RSV-group infections and clinical severity. A number of studies were carried out but such a relationship has not been fully elucidated [12].

Although RSV has been recognized as an important pathogen in childhood, there is no published information regarding the molecular epidemiology and clinical characteristics of RSV infections in Vietnam. The aims of this study were to investigate the molecular epidemiology of RSV infections, as well as to compare the clinical characteristics of diseases caused by group A and B strains in hospitalized children in Ho Chi Minh City, Vietnam.

## Materials and Methods

### Patients and samples

The study was conducted from April 2010 to May 2011 at the Respiratory Ward, the Children's Hospital 2, in Ho Chi Minh City, Vietnam. The Children's Hospital 2 is a 1,000-bed tertiary referral and university-affiliated hospital, receiving pediatric patients from most parts of the city as well as other provinces in the south of Vietnam. This area has a tropical climate with two distinct seasons: rainy season (May–October) and dry season (November–April). The daily temperature does not change much during the year conducting this study.

Under 15-year-old children admitted for an ARI condition with an onset of illness less than 7 days were enrolled. The study was approved by the Scientific and Ethical Committee of the Children's Hospital 2. The written consent was obtained from the parent or legal guardian of the participants. An ARI case was defined as any child presenting with cough and/or difficult breathing [13]. Patients who had underlying chronic diseases (e.g. cystic fibrosis, bronchopulmonary dysplasia, congenital heart disease, immunodeficiency) or who were discharged from the hospital within the previous 7 days, or who had coexisting acute systemic illnesses (e.g. sepsis), or proven or suspected non-infectious respiratory symptoms (e.g. asthma), were all excluded from the study.

Demographic and clinical data were recorded on a standardized questionnaire. The diagnosis was made on the basis of clinical findings and chest X-ray (CXR). ARI patients with the presence of an infiltrate on CXR were categorized as pneumonia. Bronchiolitis was defined as ARI patient under 2 years old presenting with wheezing and hyperaeration, atelectasis, or peribronchial thickening on CXR. Croup was characterized by hoarseness, cough, and stridor. URTI was defined as ARI with no abnormalities on CXR. The disease severity was assessed by using the previously published severity score [14], [15].

Nasopharyngeal flocked swabs (MicroRheologics, Brescia, Italy) were obtained by trained personnel on 2 fixed days each week from all enrolled children within 24 hr after admission. The specimens were immediately placed in tubes containing 2 ml sterile physiological saline fluid and stored at −20°C until further analysis at the laboratory.

### Virus detection

Viral genomes were extracted directly from the specimens by using the QIAamp Viral RNA Mini Kit (Qiagen, Hilden, Germany) according to the manufacturer's instructions and stored at −80°C.

All specimens were screened for RSV and other respiratory viruses such as influenza virus A and B, human metapneumovirus, parainfluenza virus types 1 to 4, human rhinoviruses (HRV), human coronaviruses (229E and OC43), adenovirus and human bocavirus by using multiplex hemi-nested (RT)-PCR as described previously [16]. The detection primers were targeted to the conserved region of nucleoprotein gene of RSV.

### Genotyping of RSV

All samples positive for RSV by screening test were then subjected to grouping and genotyping by a hemi-nested PCR as described previously [8]. The second hypervariable region of the G protein gene of RSV was the target for the outer and inner PCRs. The final product sizes were possible for differentiating RSV group A, group B, and genotype BA with each other. The genotypes within each group were further identified by sequence analysis.

The products of nested PCR were sequenced bi-directionally by the commercial company (Macrogen Japan Corp., Tokyo, Japan). The nucleotide sequences were analyzed and compared with the reference strains available in the GenBank database. The sequence data and the phylogenesis were analyzed using BioEdit v.7.0.5 [17]. A parsimony analysis was also conducted using MEGA version 3.1 [18]. The method was performed using close-neighbor interchange with a random option and with 1,000 bootstrap repetitions.

The sequences of RSVs detected in this study have been submitted to GenBank and assigned accession numbers JX079948–JX079993.

### Statistical analysis

Values were given as percentages for categorical variables, and as median with range for continuous variables. Categorical variables were compared by using χ^2^ test or Fisher's exact test, and continuous variables were compared by using the Mann-Whitney *U* test. A two sided value of p<0.05 was considered statistically significant. All analyses were conducted using the Statistical Package for Social Sciences version 16.0 (SPSS, Inc., Chicago, IL, USA).

## Results

From April 2010 to May 2011, 1,082 cases of ARI were enrolled in this study. The median age was 9 months (ranged from 0 to 161 months), 86% of patients were under 2 years old. The boy to girl ratio was 1.8∶1, indicating that boys were more commonly affected than girls.

### RSV detection and seasonal pattern

**Figure 1:**
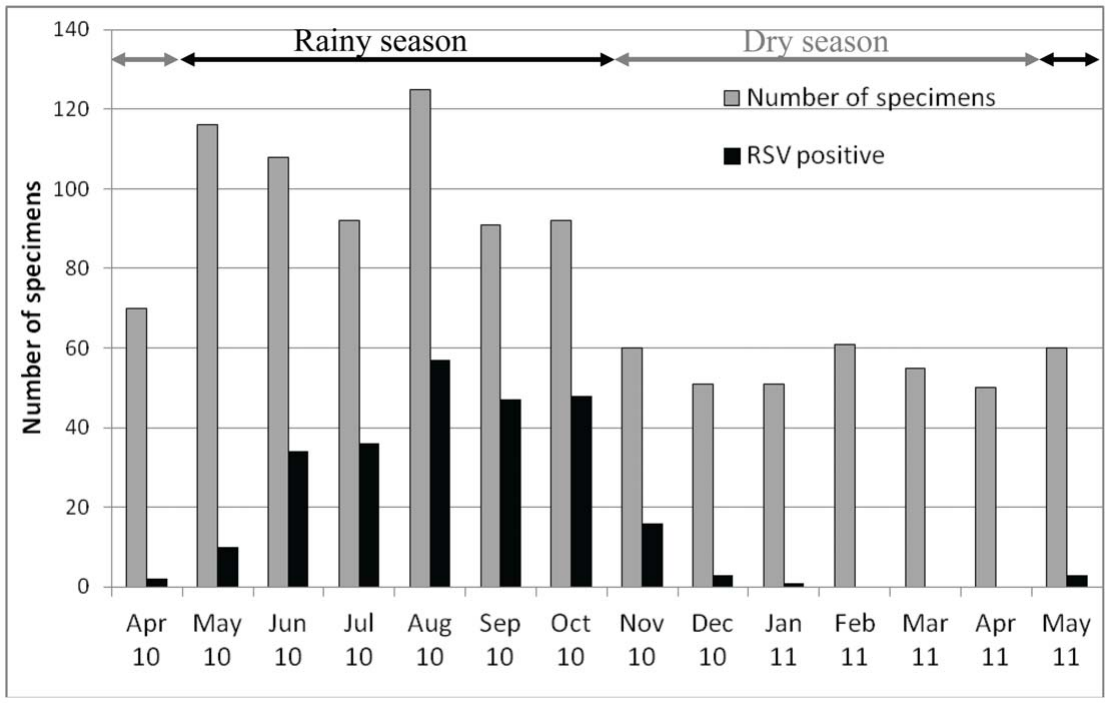
Monthly distribution of RSV infections from April 2010 to May 2011.

One or more respiratory viruses were identified in 63.8% (690/1,082) of patients, including 23.8% (257/1,082) that were positive for RSV. RSV was the second most common virus following HRV. Additionally, 23% (59/257) of all RSV positive samples contained at least one other respiratory virus. Co-infection between RSV and HRV was the most frequent (51 cases). RSV A was the dominant subgroup with 91.4% (235/257). RSV subgroup B had 5.1% (13/257). Interestingly, 9 cases (3.5%) contained both subgroup A and B RSV. The RSV epidemic occurred during the rainy season, from May to October (Figure 1).

### Molecular epidemiology of RSV

**Figure 2:**
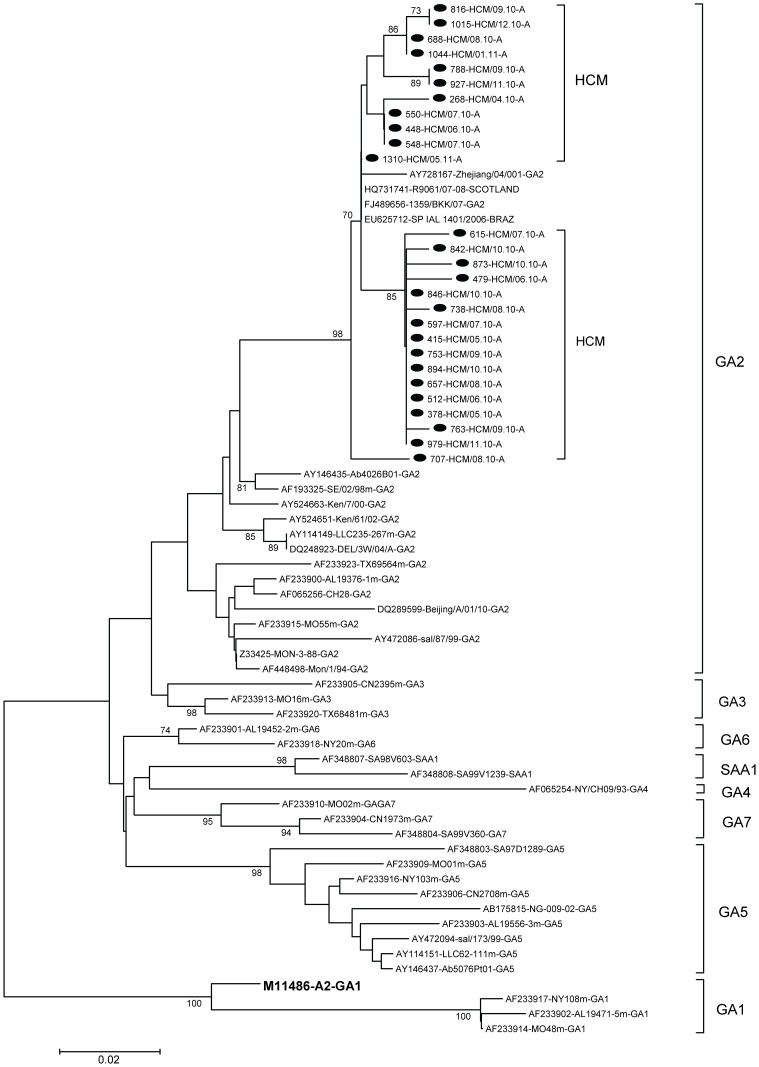
Phylogenetic tree for RSV-A nucleotide sequences based on 2^nd^ variable region of G gene. Phylogenetic tree was constructed with MEGA 3.1 software using the neighbor-joining method. Bootstrap values of greater than 70% are shown at the branch nodes. The RSV strains in this study are marked with solid round. Prototype strain A2 (in bold face) for group A was also included. The genotype assignment is indicated by the brackets on the right.

Twenty-seven RSV A and 19 RSV B samples were selected randomly for sequencing. Twenty-seven of the group A strains clustered into one genotype, GA2 (Figure 2). Of these, 9 sequences were found only once. Of the remaining 18 sequences, 5 sequence groups were found, with 2 to 9 isolates per group. The rates of divergence between prototype strain A2 and the Vietnamese strains were 12.2% to 14% at the nucleotide level and 24.2% to 28.8% at the amino acid level. Differences of up to 3.2% at the nucleotide level and 7.6% at the amino acid level were observed among the group A Vietnamese strains. The nucleotide and amino acid distances between the Vietnamese strains and the GA2 reference strains were up to 9% and 21.2%, respectively. The G protein gene of one of these strains, 1310-HCM/05.11-A, was identical to three sequences in GenBank, from Brazil (EU625712-SPIAL 1401/2006), Thailand (FJ489656-1359/BKK/07), and Scotland (HQ731741-R9061/07-08).

**Figure 3:**
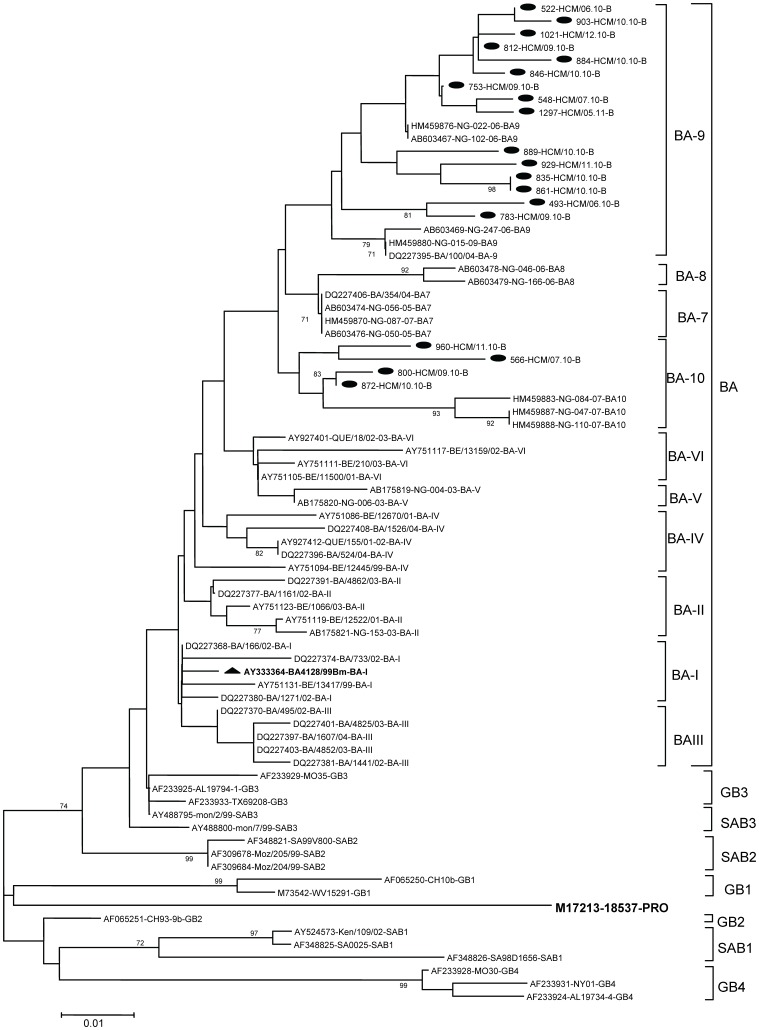
Phylogenetic tree for RSV-B nucleotide sequences based on 2^nd^ variable region of G gene. Phylogenetic tree was constructed with MEGA 3.1 software using the neighbor-joining method. Bootstrap values of greater than 70% are shown at the branch nodes. The RSV strains in this study are marked with solid round. Prototype strain of BA genotype is marked with solid triangle. Prototype strain 18537 for group B was also included. The genotype assignment is indicated by the brackets on the right.

All 19 of the group B strains belonged to the recently identified BA genotype, with a 60-nucleotide duplication in the second variable region of the G protein gene [10]. These BA genotype strains were further clustered into 2 recently described subgenotypes BA9 and BA10 [19] (Figure 3). The nucleotide and amino acid variations among the Vietnamese BA genotype strains were up to 7% and 12.9%, respectively. There was 2.5% to 6% divergence at the nucleotide level and 4.8% to 11.3% divergence at the amino acid level between the Vietnamese strains and the prototype genotype BA. Regarding the BA9 subgenotype, differences of up to 6% at the nucleotide level and 8.1% at the amino acid level were observed when comparing Vietnamese strains with reference strains. The nucleotide and amino acid distances between Vietnamese BA10 strains and BA10 reference strains were up to 5.5% and 11.2%, respectively.

**Figure 4:**
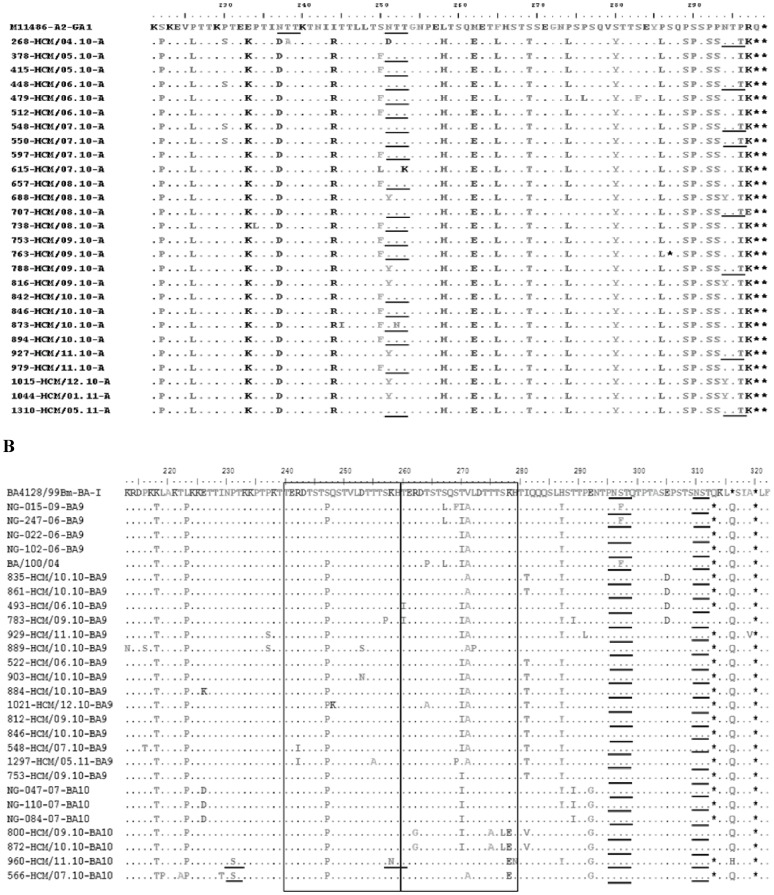
Amino acid alignments of 2^nd^ variable region of G protein from RSV-A (A) and RSV-B (B). Alignments are shown relative to the sequences of prototype strain A2 (GenBank accession number M11486) (A) and genotype BA strain BA4128/99B (GenBank accession number AY333364) (B). The amino acids shown correspond to strain A2 G protein positions 221 to 299 for the group A viruses or to strain BA4128/99B G protein positions 213 to 320 for the group B viruses. Identical residues are indicated by dashes. The two copies of the duplicated 20-amino-acid region in group B viruses are indicated by rectangles. Stop codons are indicated by asterisks. Potential N-glycosylation sites (NXT, where X is not proline) are underlined.

The deduced amino acid sequences of the group A and group B isolates were compared to those of the prototype A2 and BA strains, respectively (Figure 4). Twenty-seven of the Vietnamese group A viruses exhibited changes in the stop codon position compared with that of prototype strain A2, which had 298 amino acids in the deduced G-protein sequence. Among these, 26 Vietnamese group A strains had a predicted G protein of 297 amino acids, while 1 strain (763-HCM/09.10-A) had a premature stop codon that shortened the G polypeptide to 286 amino acids.

The G protein genes of the BA genotype were predicted to encode proteins of 2 different lengths, 312 (16/19 isolates) and 319 (3/19 isolates) amino acids. A serine (S) to proline (P) substitution was found within the 20-amino acid duplicated region at position 247 in all the Vietnamese BA strains. In addition, 14 of the 15 BA9 strains had a V271A substitution relative to the BA prototype strain, whereas all 4 BA10 strains had an E292G change. The majority (11/15) of the BA9 isolates also had amino acid substitutions at positions 270 (T270I) and 287 (H287Y).

The G protein is heavily glycosylated with both N-linked and O-linked sugars. Two putative N-glycosylation sites (NXT, where X is not proline) were identified in the second variable region of the G protein among the majority of the RSV A strains. The amino acid substitutions at some positions made these strains gain or lose the N-glycosylation sites. Similarly, two N-glycosylation sites were found to be conserved in all BA strains examined. Two of the BA10 isolates, 960-HCM/11.10-BA10 and 566-HCM/07.10-BA10, each acquired an additional N-glycosylation site as a result of amino acid substitutions at P231S and K258N, respectively.

### Disease severity of RSV infections

To examine the disease severity in the RSV positive and negative groups, clinical and demographic data were compared (Table 1). With regard to age, RSV positive children were significantly younger than RSV negative ones (7 vs. 11 months, *p*<0.001). RSV positive patients were admitted to the hospital earlier in the course of their disease than RSV negative patients (2 vs. 3 days, *p* = 0.017). They also had higher rates of fever (72.8 vs. 65.5%, *p* = 0.033), runny nose (82.5 vs. 70.5%, *p*<0.001), and lower chest indrawing (68.5 vs. 51.0%, *p*<0.001). Most importantly, the clinical severity score of the RSV positive children was significantly higher (12 vs. 11, *p* = 0.003) than the RSV negative group. Regarding the diagnosis, RSV positive patients were more likely to have bronchiolitis (47.1 vs. 28.8%, *p*<0.001).

**Table 1:** Demographic and clinical characteristics associated with RSV-positive, negative, mono-, co-infection and subgroups.

Characteristics (%)	RSV pos N = 257	RSV neg N = 825	p	RSV mono N = 198	RSV co N = 59	p	RSV A N = 177	RSV B N = 12	RSV A&B N = 9	p
Male	63	65.2	NS	63.6	61	NS	64.4	50	66.7	NS
Age (m)^a^	7(3–14)	11(4.5–19)	**<0.001** c	7.5(3–14)	6(2–13.5)	NS	7 (3–14.5)	7.5 (3–13.5)	8 (2–11)	NS
Age group										
<6 m	42.8	29.9	**<0.001** b	41.4	47.5	NS	41.7	41.7	44.4	NS
6–<12 m	26.5	23.2	NS	28.3	20.3	NS	27.8	16.7	44.4	NS
12–<24 m	20.2	29.6	**0.003** b	19.7	22.0	NS	18.9	41.7	11.1	NS
≥24 m	10.5	17.3	**0.008** b	10.6	10.2	NS	11.7	0	0	NS
Prematurity (<37 weeks)	10.9	8.6	NS	10.7	11.9	NS	10.6	16.7	0	NS
Malnutrition	6.2	10.5	**0.039** b	7.1	3.4	NS	6.1	25	0	RSV A v RSV B p = 0.046^b^
Infection with other viruses	23.0	52.5	**<0.001** b	NA	NA		NA	NA	NA	
Days before hos.^a^	2(2–4)	3(2–5)	**0.017** c	2(2–4)	3(2–4)	NS	2 (2–4)	2 (1–2.5)	3 (3–5)	RSV B v RSV AB p = 0.023^c^
Fever	72.8	65.5	**0.033** b	74.7	66.1	NS	73.3	83.3	100	NS
Cough	93.8	89.9	NS	93.4	94.9	NS	92.8	100	100	NS
Runny nose	82.5	70.5	**<0.001** b	84.3	76.3	NS	83.9	83.3	100	NS
SpO_2_≤92%	5.8	9.3	NS	4.5	10.2	NS	4.4	8.3	0	NS
Tachypnea	41.6	45.5	NS	42.9	37.3	NS	44.4	41.7	11.1	NS
Lower chest indrawing	68.5	51.0	**<0.001** b	69.2	66.1	NS	70	50	77.8	NS
Wheezing^d^	59.9	58.5	NS	59.6	61	NS	60.6	25	88.9	RSV A v RSV B p = 0.03^b^; RSV B v RSV AB p = 0.014^b^;
Clinical Severity Score^a^	12(8–13)	11(7–13)	**0.003** c	12(8–13)	12(8–13)	NS	12 (8–13)	8 (7.5–12)	13 (8–14)	RSV A v RSV B p = 0.031^c^; RSV B v RSV AB p = 0.028^c^;
Diagnosis										
URTIs	16.0	23.4	**0.012** b	17.7	10.2	NS	18.9	8.3	0	NS
Croup	0.8	7.8	**<0.001** b	1	0	NS	1.1	0	0	NS
Bronchiolitis	47.1	28.8	**<0.001** b	44.9	54.2	NS	42.8	66.7	66.7	NS
Pneumonia	36.2	40.0	NS	36.4	35.6	NS	37.2	25	33.3	NS
Hos. duration^a^	6(4–8)	5(4–8)	NS	6(4–8)	6(4–7.5)	NS	6 (4–8)	7 (5–8)	6 (3–6)	NS

Abbreviation: d, day; m, month; hos, hospitalization; URTI, upper respiratory infection; RSV, respiratory syncytial virus; NA, not applicable; NS, not significant; pos, positive; neg, negative; mono, mono-infection; co, co-infection.

Note: All results are expressed in percentages except for (^a^) in median with interquartile range between brackets.

b Chi-squared test.

c Mann-Whitney-*U* test.

To address the question of whether children with RSV co-infection have different characteristics to those with RSV mono-infection, attempts were made to compare these two groups (Table 1). However, differences were not statistically significant.

To assess the relationship between clinical severity and RSV subgroup, subjects that were co-infected with other viruses were excluded. The 177 cases with RSV A, 12 cases with RSV B, and 9 cases with mixed infection of both RSV A and B were compared (Table 1). No significant differences were found with respect to the presence of fever, cough, runny nose, tachypnea, lower chest indrawing, and hypoxemia among the three groups. However, both wheezing and the clinical severity scores were greater in patients with group A and mixed A and B infection than those with group B infection (*p*<0.05).

In multivariate analysis adjusted for age, sex, prematurity, malnutrition and infection with other viruses, the difference on days before hospitalization and on the clinical severity score remained significant between the RSV positive and negative group (*p*<0.001 and <0.001, respectively). Children infected with group A virus still had a significantly higher clinical severity score than those infected with group B (*p* = 0.049) but those with mixed infection of both groups did not (*p* = 0.064).

## Discussion

RSV is one of the most important respiratory pathogens among infants worldwide. The identification of RSV in about one fourth of cases in this study confirmed that this virus is a dominant agent of respiratory disease in children. This finding is in line with previous reports from Vietnam and other countries [16], [20], [21], [22], [23], [24].

Regarding the seasonality in this study, the RSV epidemic peaked during the rainy season. Interestingly, RSV activity was completely absent for the 3 months of the dry season (February to April, 2011). The presence of RSV infection seasonality in relation to rainfall has been observed in India [25], Hong Kong [26], Thailand [27], the Philippines [27], Colombia [28], and Kenya [29]. In tropical regions, children tend to be kept indoors during the rainy season, and the resultant crowding may account for the increased incidence of RSV infection. Another reason that has been suggested is that high humidity may help to prevent the virus from desiccation and loss of infectivity.

RSV is the most common viral pathogen causing lower respiratory tract infections (LRTIs) among infants and young children. However, there has been no information about the molecular epidemiology of RSV in Vietnam. This report provides data on the molecular characteristics of RSV from hospitalized children in Ho Chi Minh City and represents the first such study in Vietnam. Information from this study will contribute to the growing database on the molecular diversity of RSV circulating worldwide. The results of this study indicated that RSV subgroup A and B co-existed in one epidemic and cases of RSV subgroup A infection predominated over those of subgroup B. These findings are in agreement with majority of studies in many countries around the world including Germany [30], Belgium [31], Argentina [2], Kenya [32], Japan [33], and India [8], with various patterns of subgroup predominance.

The identification of the GA2 as the predominant genotype in this study was consistent with the results of other reports. GA2 was the most common genotype of RSV group A found around the world and has persisted for many years [8], [30], [31], [33], [34]. On the other hand, the G protein gene variable region of one Vietnamese strain was identical to strains from different parts of the world, including Thailand, Scotland, and Brazil.

In this study, 26/27 of GA2 genotype strains were predicted to encode a G protein of 297 amino acids in length, while one strain was shorter, of 286 amino acids. This mutant strain was sequenced directly from the clinical sample. To our knowledge, there has been one previous report of such a mutant strain, by Cane and Pringle [35]. In that report, an isolate with the G protein of 289 amino acids was obtained from virus culture. Our strain was genotype GA2. The previously reported strain was genotype GA1 (data not shown).

Surprisingly, all Vietnamese subgroup B viruses fell into a new genotype BA, which was first detected in Buenos Aires, Argentina during 1999 [10]. The major characteristic of genotype BA is that the G protein gene contains a 60-nucleotide duplication in the second variable region. The BA genotype strains in this study were further clustered into 2 recently described subgenotypes, BA9 and BA10. These subgenotypes were first described from RSV isolates in Japan in 2006 [19] but have not been reported elsewhere until now.

The Vietnamese BA strains had two different G protein lengths, 312 and 319 amino acids, which were reported in previous studies [36]. In addition, alterations had occurred so that the duplicated region was no longer identical to the original one. Since its first appearance, the BA genotype has spread globally and was reported from many regions around the world. Some recent reports also showed that genotype BA has gradually replaced the other group B genotypes [10]. It is possible that these changes in the G protein enhance the attachment of the virus to the host cell, or result in the antigenic modification which allows this virus to escape the immune response.

Potential N-glycosylation sites are thought to play an important role in helping viruses escape from the host immune response [37]. The number and distribution pattern of glycosylation sites identified in this study were different between the 2 RSV subgroups. Our group A strains had 2 N-glycosylation sites within the second variable region of G protein while group B had 4 (Figure 4). Variations in the number and location of these sites can inhibit the recognition of RSV by antibodies to particular epitope [38].

The demographic and clinical information is important to put the results into a practical context and allow them to be applied in clinical practice. In the current study, statistical significance was achieved for the detection of RSV and its association with clinical severity. The RSV-positive patients tended to have more severe symptoms and a higher severity score that probably led them to seek hospital care earlier than the RSV-negative patients.

The fact that nearly half of the RSV infections (42.8%) occurred in children under 6 months showed that patients in this age group are the most vulnerable to RSV infections, despite the presence of maternal antibodies. Maternal antibodies are able to protect against severe RSV in children, however relatively high tilters are required [39]. Unfortunately, with a half-life of 26 days antibodies received at birth quickly drop to unprotective levels within the first month of life [39]. The finding that RSV infections were mainly associated with bronchiolitis and pneumonia (47.1 and 36.2% of the cases, respectively), in which the diagnosis of bronchiolitis was significantly related to RSV, confirmed that this agent is an important cause of LRTIs, as have other studies [40], [41].

The detection of co-infection has led to the speculation that the presence of several types of virus in one respiratory specimen may affect the clinical presentation of ARIs. Viruses might interact indirectly or directly, and the effect may depend upon which viruses are co-infecting. The combination of hMPV and RSV has been associated with increased disease severity requiring the use of mechanical ventilation [42], [43]. No additional effect of mixed infection of HRV and RSV on disease severity was observed [41]. In the present study, no relationship between RSV co-infections and increased disease severity was established.

Regarding clinical manifestation of each group, by using a composite severity score, we found that infections involving RSV group A (alone or mixed with RSV group B) were associated with more severe disease than infection with RSV group B. Besides virus factors, the clinical severity of RSV infection is also associated with epidemiological and host factors, which include socioeconomic status, age, prematurity, and underlying heart and/or lung disease. To exclude these confounding factors, patients with underlying diseases or having co-infection with other viruses were not included.

Subgroup B infected children were admitted to the hospital less frequently than subgroup A infected children. Either subgroup B strains cause such a mild illness that there is no need to hospitalize the patient or the prevalence of subgroup B is truly low. However, in two studies, the proportions of subgroup A and subgroup B infected children in the hospital and in the community were similar [44], [45].

Several studies have examined the relationship between clinical severity and RSV subgroups. In approximately half of these studies, group A seemed to be associated with more severe clinical disease [44], [45], [46], [47], [48], [49], [50], whereas no such difference was found in the others [11], [51], [52], [53], [54], [55], [56]. In only two studies have group B infections been reported to cause more severe disease [57], [58]. This inconsistency could be attributed to difference in study design and population, definition of disease severity, the distribution of RSV subgroups, etc. In the recent study, Houben *et al.* reported that disease severity correlated positively with viral load during primary RSV infection [15].

The current study was limited to only one epidemic season. Because the predominant subgroups shift from year to year, the immunity against the previous circulating groups could have altered the severity of disease caused by specific subgroups. Therefore, continued observation and analysis of additional seasons is required to determine the association between subgroups and severity of disease.

In summary, RSV was found to be the viral pathogen most commonly and frequently associated with severe acute respiratory diseases in infants and children. Co-infections between RSV and other respiratory viruses did not lead to increased disease severity. The molecular characteristics of RSV were determined for the first time in Vietnam, and carried characteristics both similar to strains from other parts of the world and specific to Vietnam. Subgroup A and B of RSV were co-circulating and subgroup A caused more severe disease than subgroup B. Similar surveillance should be continued to follow the epidemiology of this virus. A better understanding of RSV epidemiology is essential to enable prediction of outbreaks and for planning preventive and therapeutic control measures.
